# Whole Transcriptome Analysis of Obese Adipose Tissue Suggests u001kfc.1 as a Potential Regulator to Glucose Homeostasis

**DOI:** 10.3389/fgene.2019.01133

**Published:** 2019-11-21

**Authors:** Linlin Yang, Xing Wang, Huaibin Guo, Wanxing Zhang, Wei Wang, Huijuan Ma

**Affiliations:** ^1^Clinical Medicine Research Center, Hebei General Hospital, Shijiazhuang, China; ^2^Department of General Surgery, Hebei General Hospital, Shijiazhuang, China; ^3^Department of Pediatrics, The Fifth Hospital of Shijiazhuang, Shijiazhuang, China; ^4^Department of Endocrinology, Hebei General Hospital, Shijiazhuang, China

**Keywords:** obesity, long non-coding ribonucleic acid, network, glucose homeostasis, adipose tissue

## Abstract

Long non-coding RNA (LncRNAs) are newly highlighted key factors controlling brown adipogenesis and development, but their regulatory effect to white adipocyte is still merely understood. Deciphering their underlying mechanism could be a novel way to discovering potential targets of obesity. Therefore, we conducted a whole transcriptome analysis in white adipose tissue from obese patients for the first time. Six obese patients and five control subjects were selected for microarray assay. Differentially expressed coding genes (DEGs), targets of lncRNAs, and alternatively spliced genes in obesity group were systematically compared in a functional framework based on a global gene regulatory network. It was demonstrated that all the three kinds of transcripts were enriched in pathways related to glucose metabolism while only DEGs showed closer proximity to neuro-endocrine-immune system. Thus, a lncRNA-regulated core network was constructed by a stepwise strategy using DEGs as seed nodes. From the core network, we identified a decreased lncRNA, uc001kfc.1, as potential *cis*-regulator for phosphatase and tensin homolog (PTEN) to enhance insulin sensitivity of white adipocytes in obese patients. We further validated the down-regulation of uc001kfc.1 and PTEN in an independent testing sample set enrolling 22 subjects *via* qRT-PCR. Although whether the decreased uc001kfc.1 correlated with low risk of diabetes deserved to be examined in an expanded cohort with long-term follow-up visit, the present study highlighted the potential of lncRNA regulating glucose homeostasis in human adipose tissue from a global perspective. With further improvement, such network-based analyzing protocol proposed in this study could be applied to interpreting function of more lncRNAs from other whole transcriptome data.

## Introduction

Over the past decade, obesity has become a global health-threatening disease resulting from unbalanced energy intake and expenditure. Except for the uncontrolled body weight, obesity often accompanied by complications such as type 2 diabetes, fatty liver, and cardiovascular diseases in a long-term and chronic manner (Boles et al., 2017; Vecchie et al., 2018). Although several adipokines, cytokines and related pathways have been implicated in obesity, its exact mechanism still has not been elucidated yet.

Long non-coding RNA (lncRNA) is defined as transcript ≥ 200 nt with low protein-coding potential. It has been shown to drive numerous biological processes such as cell development, differentiation, and metabolism since it was discovered. Recently, it was linked to adipogenesis and cell differentiation in white and brown adipocyte (Xiao et al., 2015; Xiong et al., 2018). Based on mouse model, it was found that lncRNA could dynamically regulate energy homeostasis (Bai et al., 2017), control brown adipocyte differentiation (Zhao et al., 2014), and maintain its morphology (Alvarez-Dominguez et al., 2015). Besides, several lncRNAs recognized from human adipose tissue were characterized to be related to adipogenesis (Ding et al., 2018). For example, Smith *et al*. examined ectopic expression of HOTAIR and found that it could promote differentiation of human gluteal preadipocytes (Divoux et al., 2014). Zhou *et al*. observed down regulation of MEG3 during adipogenesis and confirmed its role in human adipogenic differentiation *via* knockdown assay (Li et al., 2017).

Although the above insightful studies have shown the essential role of lncRNAs in fat biology, to interpret their mechanisms in obesity, a systematical analysis of all transcripts is still urgently needed because they are the base for lncRNAs’ function implementation. The widespread effects of lncRNA on gene transcription include but are not limited to shaping chromosome conformation, recruiting transcription factors (Marchese et al., 2017), and even indirectly targeting downstream genes by interacting with other non-coding RNAs (Cesana et al., 2011). As a reflection of this delicate progress, whole transcriptome provides a reliable and effective way to uncovering latent mechanism of lncRNA in obesity.

In the present study, the whole transcriptome of adipose tissues collected from obese individuals and control subjects were analyzed. A stepwise network reconstruction strategy was proposed to explore lncRNA-related functional modules altered in obesity. A decreased lncRNA named uc001kfc.1, which was recognized from the core network, was suggested to be related with enhanced insulin sensitivity of white adipocytes in obese patients by *cis*-regulating phosphatase and tensin homolog (PTEN). Our study highlighted the potential of lncRNA regulating glucose homeostasis in human adipose tissue from a global perspective, which would facilitate deeply understanding pathology of obesity as well as developing novel therapeutic targets.

## Methods

### Subject Selection and Grouping

Our study was approved by the Institutional Review Board of Hebei General Hospital and conducted according to the principles expressed in the Declaration of Helsinki. As invasive surgery was the only way to obtain adipose tissues, 33 asymptomatic cholecystolithiasis patients receiving cholecystectomy were recruited as donors. All participates had no acute symptoms induced by cholecystolithiasis such as infection, fever, and jaundice. All volunteers had not taken any anti-cholecystitis drugs such as magnesium sulfate, dehydrocholic acid, ursodeoxycholic acid, and chenodeoxycholic acid before surgery. Besides, we also excluded volunteers with medication history of weight loss drugs such as orlistat, lorcaserin, naltrexone-bupropion, phentermine-topiramate, and liraglutide. According to the recommendations of the Working Group on Obesity in China, obesity was defined as a body mass index (BMI) of at least 28 kg/m^2^ (Zhou, 2002). Thus, a total of 11 obese (BMI ≥ 28 kg/m^2^) and 22 age-matched control (BMI < 28 kg/m^2^) subjects were enrolled in this study. Visceral fat tissues from donors were collected during cholecystectomy and immediately stored at −80°C until microarray assay. The use of these subjects was approved by the hospital’s Ethics Committee and all participants provided their written informed consents.

To evaluate the reliability of potential findings based on whole transcriptome analysis, a training, and testing strategy was used for sample grouping. We assigned the collected subjects into two independent sample sets: discovery set and validation set. Totally, six obese patients and five control subjects were randomly assigned to the discovery set, which was used for microarray to high-throughput screen potential transcripts critical to obese adipose functions. The remaining samples were assigned to the validation set which was designed to independently validate the identified transcripts.

### Microarray Assay

The whole transcriptome profiles of subjects in microarray set were detected through GeneChip^®^ Human Transcriptome Array 2.0 (HTA2.0) according to the manufacturer’s instructions. The microarray assay was performed by Shanghai Biotechnology Cooperation and the process were detailed in the attachment file (see **Supplementary Method**). The obtained microarray data was released to the National Center for Biotechnology Information (NCBI) Gene Expression Omnibus (GEO) database with accession number GSE133786.

### Principle Component Analysis

Principle component analysis of microarray data was performed through Transcriptome Analysis Console 4.0 (TAC4.0) software to assess the overall variances of selected subjects.

### Differentially Expression Analysis

To determine a robust average unaffected by outliers, we use Tukey’s biweight algorithm to compute average signal in each group (Affymetrix, 2002). Subsequently, foldchanges were calculated as formula (1).

(1) $$\log\mathrm{FC}=\;\;\bar{\mathrm{lo}g_{2}\mathrm{Biweight}\_\mathrm{Signa}l_{\mathrm{Obesity}}}-\;\;\bar{\mathrm{lo}g_{2}\mathrm{Biweight}\_\mathrm{Signa}l_{\mathrm{Control}}}$$

One-way between-subject ANOVA was used to determine the significance of difference between obesity and control group in term of P-value. Genes with |logFC| ≥ 1.5 and P-value < 0.05 were regarded to be differentially expressed between groups.

### Assessment of Alternative Splicing Events

Here, we assessed alternative spliced genes based on transcripts from exons, which were provided by HTA2.0 microarray. Splicing index (SI) was used to compare the relative intensity of each exon between two groups. SI for exon *i* in gene *j* was calculated as

(2) $$SI_{\mathrm{Exon}\;i}=\;\frac{\mathrm{Intensit}y_{\mathrm{Exon}\;i,\;\;\;\mathrm{Obesity}}/\mathrm{Intensit}y_{\mathrm{Gene}\;j,\;\;\;\mathrm{Obesity}}}{\mathrm{Intensit}y_{\mathrm{Exon}\;i,\;\;\;\mathrm{Control}}/\mathrm{Intensit}y_{\mathrm{Gene}\;j,\;\;\;\mathrm{Control}}}$$

One-way between-subject ANOVA was used to determine each exon’s significance of difference between obesity and control group in term of P-value. Genes with |SI| ≥ 2 and P-value < 0.05 were regarded to be alternatively spliced in obesity group. This process was performed by TAC 4.0 software. Additionally, TAC provides annotation for alternatively spliced genes based on “how well the data fits into pre-defined splicing pattern,” which is measured by “Exon Event Estimation Score” (EEES). Accordingly, alternatively spliced genes with EEES > 0.2 were labeled by exon events such as intron retention, cassette exon, alternative 3’ acceptor site, alternative 5’ donor site, and mutually exclusive exons.

### Prediction of *cis*- and *trans*-Targets

Differentially expressed lncRNAs were selected for prediction of *cis-/trans*-targets. *Cis*-targets of lncRNAs were recognized by searching genes intersecting the region which stretched from 10 kb upstream of transcriptional start site to 10 kb downstream of termination site of the interested lncRNA. To classify lncRNA *trans*-target genes, the BLAST software was used to assess the impact of lncRNA binding on complete mRNA molecules. The RNAplex program was then used to identify possible *trans*-target genes of lncRNAs (e < 1E−20) (Tafer and Hofacker, 2008).

### Pathway Enrichment Analysis

In this paper, p-value is defined by the hypergeometric cumulative distribution function (see **Supplementary Method**) to measure the significance of candidate genes co-existing in the same Kyoto Encyclopedia of Genes and Genomes (KEGG) pathway. A cutoff of P < 0.05 means that a pathway is significantly enriched by differential or alternatively spliced genes. As there were few lncRNAs annotated in pathway database, their predicted *cis*- and *trans*-targets were used instead.

### Reconstruction of Human Gene Regulatory Network

Here we constructed a human gene regulatory network based on KEGG pathway database as our background network (Kanehisa et al., 2017). We extracted all the genes and their interactions from the *.kgml file of each human pathway in KEGG. The obtained genes were afterwards connected into an undirected network which contained 5,607 nodes and 51,748 edges.

### Proximity Parameters

Connectivity and distance are two main parameters commonly used to measure the proximity between two genes in a network. The former is defined as whether there is at least one path bridging two genes, which could measure how closely they are connected. And the latter is defined as the length of the shortest path between two genes, denoting how far they are away from each other. In this paper, the proximity between two groups of genes was calculated by the average connectivity/distance of each gene pair across two groups as previously described (Yang et al., 2012).

### Generating Core Network of Obese Adipose Tissue

To deeply exploring mechanism hidden behind the adipose transcriptome, a core network of obesity was generated. Candidate genes were introduced in the core network step by step. Firstly, differentially expressed genes were regarded as seed nodes. The relations between seed nodes and their neighbors were extracted from the background network. Then the extracted subnet was simplified by Steiner minimal tree algorism, which helped to cut unnecessary branches mainly composed of non-differential genes and keep important nodes bridging seed genes (Klein and Ravi, 1995). Subsequently, differential lncRNAs were linked to their targets in the simplified subnet. Finally, as the function of a network was mainly carried out by the most connected component, the largest component of the minimized network was extracted as the core network of obese adipose tissue. To better interpret the obtained core net, it was decomposed by simulated annealing algorithm which helped to divide the network into several modules representing different biological functions (Guimerà and Amaral, 2005a; Guimerà and Amaral, 2005b).

### Quantitative Real Time Polymerase Chain Reaction

Total RNA was isolated from adipose tissues by using TRIzol reagents (Thermo Fisher, Life Technologies, USA) and following the manufacturer’s instructions. Total RNA was quantified by NanoDrop 2000 Spectrophotometer (Thermo Fisher Scientific Inc., USA). Complementary DNA (cDNA) was synthesized by Fast Quant cDNA Synthesis Kit (TIANGEN, China). Quantitative PCR analysis were performed on ABI7500 Real-Time PCR Detection System (Thermo Fisher, USA) with SuperMix Real PreMix Plus (SYBR green) (TIANGEN, China). Sequences of primers were listed in **Supplementary Table 1**. Expression data was normalized to the geometric mean of the housekeeping gene GAPDH to control the variability in expression levels and calculated as 2 − [(CT of indicated genes) − (CT of GAPDH)], where CT represented the threshold cycle for each transcript. PCR mixtures were initially heated to 95°C for 15 min, followed by 40 cycles of 95°C for 10 s, 60°C for 20 s, and 72°C for 32 s.

## Results

### Overview of Samples and Altered Transcripts

Totally, 11 obese and 22 control subjects were enrolled in this study. The basic information of all the participants was recorded in **Table 1**. There was no significant difference of age and gender between obesity and control group. Obese subjects showed significantly higher BMI, waistline, and hipline than control group, which was consistent with the inclusion criteria. No significant differences of total cholesterol (TC), total triglyceride (TG), high density lipoprotein cholesterol (HDL-C), and low-density lipoprotein cholesterol (LDL-C) were observed between obesity and control group (P > 0.05, Student’s t test). It was unexpected that obesity group showed significantly lower fasting blood glucose (FBG) than control one (P = 0.019, Kolmogorov-Smirnov test).

**Table 1:** Demographic information of the enrolled subjects.

	Control group (*n* = 22)^§^	Obesity group (*n* = 11)^§^
Age (years)	54.82 ± 12.74	52.27 ± 13.39
Gender (male, %)	8 (36.4%)	3 (27.3%)
Waistline (cm)	85.56 ± 9.24	111.38 ± 13.61*
Hipline (cm)	95.47 ± 4.30	111.19 ± 9.79*
Height (cm)	162.59 ± 6.46	162.36 ± 6.83
Weight (kg)	63.14 ± 7.61	84.73 ± 8.52*
BMI (kg/m^2^)	23.87 ± 2.24	32.21 ± 3.54*
TC (mmol/L)	4.84 ± 1.05	4.59 ± 0.58
TG (mmol/L)	1.39 ± 1.14	1.69 ± 1.03
HDL-C (mmol/L)	1.16 ± 0.31	1.32 ± 0.70
LDL-C (mmol/L)	3.31 ± 1.01	2.84 ± 0.80
FBG (mmol/L)	5.31 (4.27–6.78)	5.11 (4.75∼10.65)^#^

^§^Information is separately presented as n (%) for dichotomous data, median (min∼max) for non-normal data, and mean ± sd for normal data.

*A significant difference between obesity and control group exists if p-value < 0.01 (two tailed Student’s t test).

^#^A significant difference between obesity and control group exists if p-value < 0.01 (two tailed Kolmogorov-Smirnov test).

**Figure 1:**
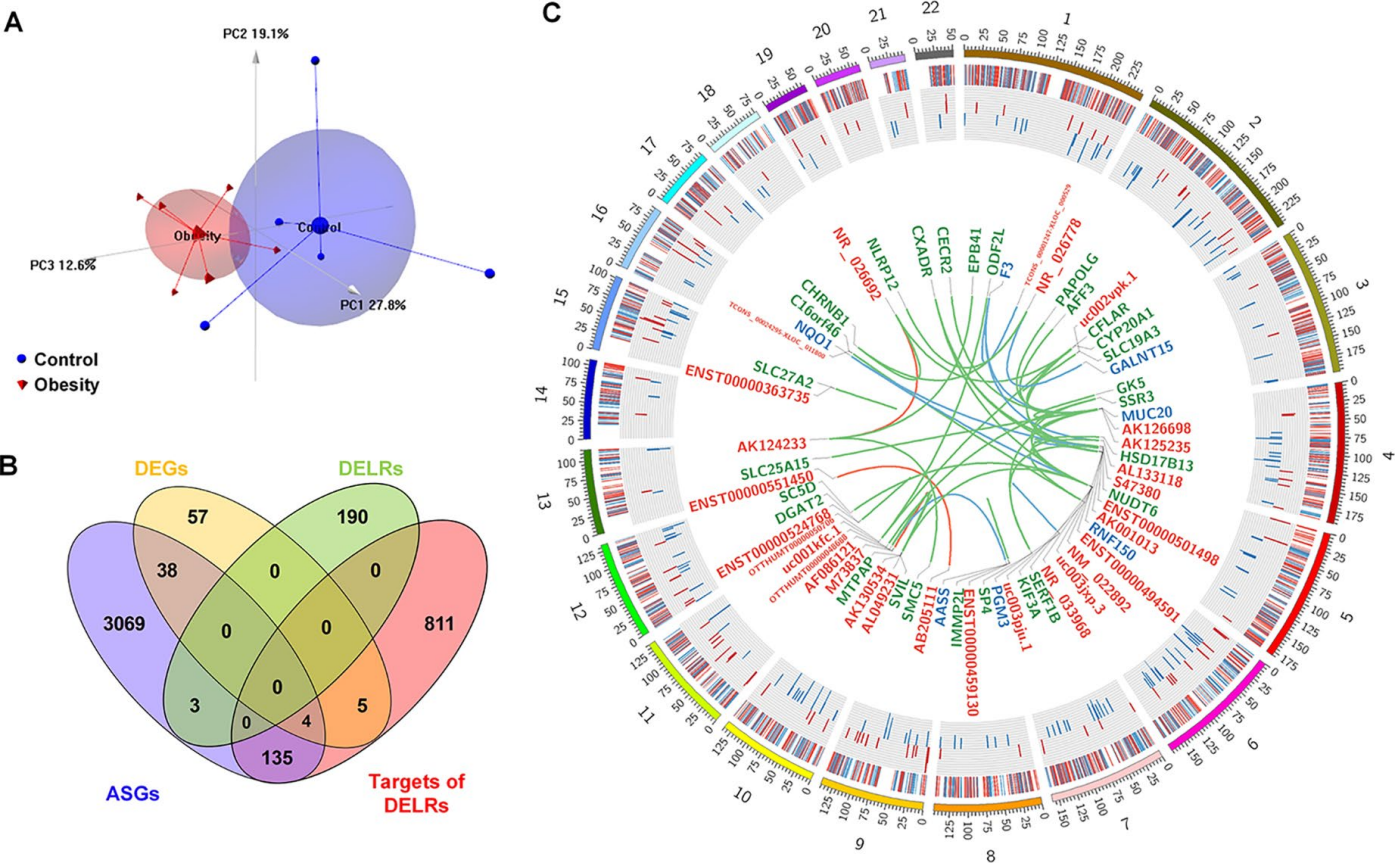
Global view of altered transcripts in obese adipose tissue. **(A)** Principle component analysis of microarray samples. **(B)** Overlapping of differentially expressed transcripts. **(C)** Chromosome distribution of altered transcripts. The heatmap of outer ring denotes fold changes of all alternatively spliced genes (ASGs); the histogram of middle ring denotes fold changes of differentially expressed coding genes (DEGs), and the links in the center denotes interactions between differential expressed long non-coding RNAs [differentially expressed lncRNAs (DELRs)] and their targets. The symbols of connected DELRs, DEGs, and annotated ASGs are respectively labeled in red, blue, and green color. Links between DELRs and these transcripts are painted by the same color as targets’ labels.

Separately, six and five subjects were randomly selected from the obesity and control group for microarray assay. Although the reduced FBG in obesity group of microarray set was not significant (P = 0.079, Kolmogorov-Smirnov test), the statistical characteristic of demographic information about these subjects remained almost the same as the original cohort (see **Supplementary Table 2**). Whole transcriptome profiles of adipose tissues from the selected samples were obtained through HTA2.0 array. As quality control, PCA was performed based on the microarray data and the top three principle component were displayed as **Figure 1A**, in which obese and control subjects were assigned into distinct regions. Based on the cutoff of |logFC| > 1.5 and P-value < 0.05, 105 coding genes and 209 non-coding ones were identified as differentially expressed transcripts (DETs). A total of 954 genes were predicted as *cis*- or *trans*-targets of the non-coding DETs. Besides, 3,421 transcripts with |SI| > 2 and P-value < 0.05 were filtered as alternatively spliced genes (ASGs), 679 of which were annotated to pre-defined exon events. Overlaps of DETs, ASGs, and predicted targets were displayed in **Figure 1B**. As it can be seen in **Figure 1B**, about 1.4% differentially expressed lncRNAs (DELRs) and 40% differentially expressed coding genes (DEGs) were alternatively spliced, indicating different transcriptional regulation mechanisms between coding and noncoding genes. **Figure 1C** showed a global view of all transcriptional alterations, including chromosome location and fold change of DETs and ASGs. Interactions between DELRs and their predicted targets were exhibited in center of **Figure 1C** as well. As it could be observed in **Figure 1C**, none chromosome was preferred by any type of the above transcripts in obese adipose tissue.

### Disturbed Pathways Suggest Aberrant Glucose Metabolism in Obesity

**Figure 2:**
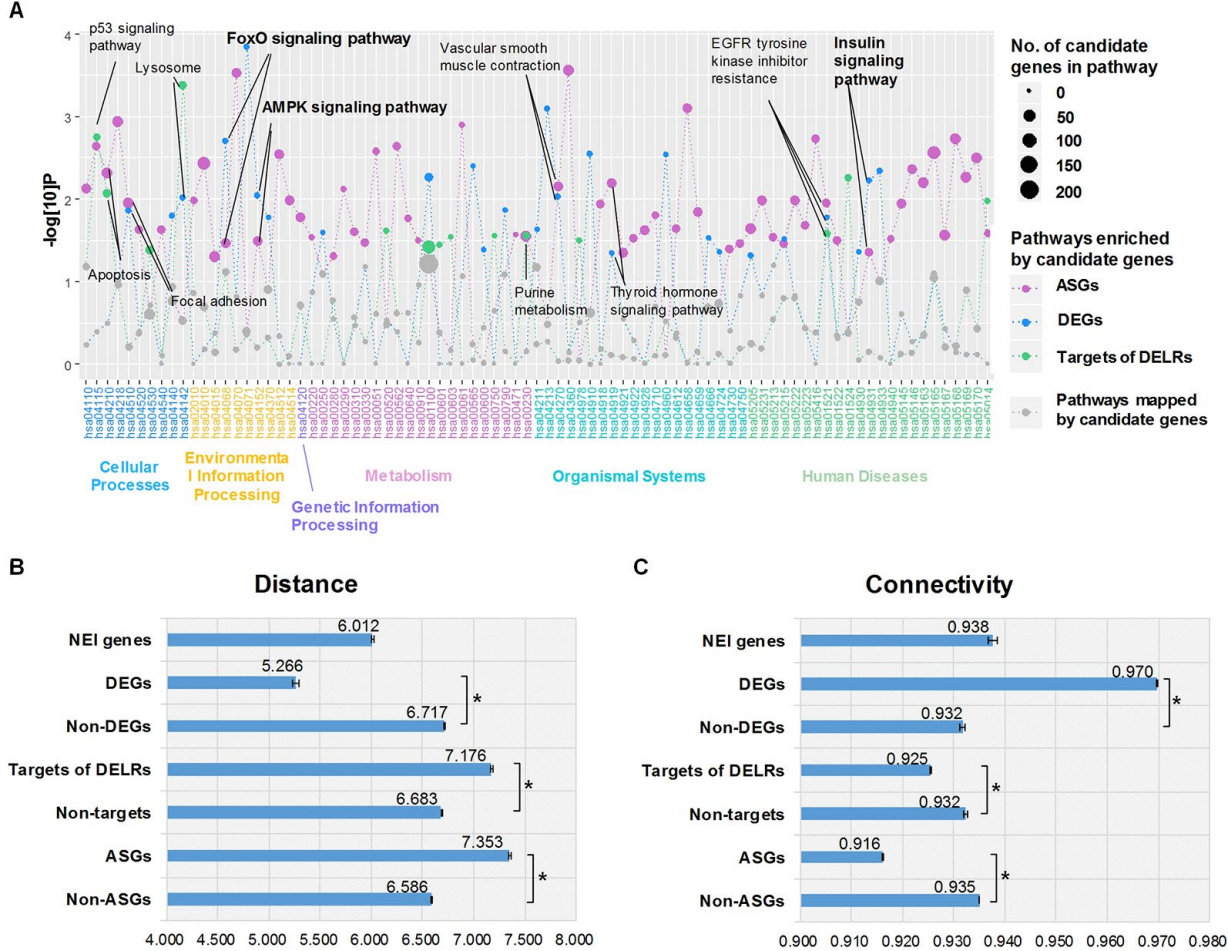
Enriched pathways and proximities to neuro-endocrine-immune (NEI) system. **(A)** Enriched Kyoto Encyclopedia of Genes and Genomes pathways of differentially expressed transcripts. **(B)** Average distances between NEI genes and altered transcripts. **(C)** Average distances between NEI genes and altered transcripts. *The distance/connectivity from NEI genes to DETs is significantly different from those to non-DETs (P < 0.001, Kolmogorov-Smirnov test).

Aiming to understand the disturbed primary biological process reflected by whole transcriptome of obesity, we respectively performed enrichment analysis for DEGs, ASGs, and targets of DELRs in KEGG pathways by using hypergeometric cumulative distribution function. **Figure 2A** displayed all the pathways mapped by any class of these transcripts. Totally, there were 11 pathways simultaneously enriched by two or more kinds of transcripts and their corresponding names were tagged by labels in **Figure 2A**. Unexpectedly, there was no specific metabolic pathway commonly enriched by the three kinds of transcripts. However, it was noteworthy that, three (27.3%) out of the 11 commonly enriched pathways, forkhead box protein O (FoxO), adenosine 5’-monophosphate activated protein kinase (AMPK), and insulin signaling pathways were typical ones regulating glucose metabolism, highlighting its significance in obese adipose tissues.

### Topological Features Indicate Closer Proximity of Obese Differentially Expressed Coding Genes to Neuro-Endocrine-Immune System

It was known that obesity was an endocrine disorder which would be systematically regulated by the neuro-endocrine-immune (NEI) system (Dixit, 2008). Thus, whether and how the altered transcripts correlate with NEI system was examined by calculating their proximity in the global gene regulatory network. According to dbNEI2.0, there were a total of 1,736 genes involved in NEI system (Zhang et al., 2008). Nine hundred forty-five of them could be mapped into our background network. Distance and connectivity between NEI genes and each kind of altered transcripts were summarized in **Figure 2**, C. Intriguingly, only DEGs showed significant shorter distance and corresponding higher connectivity to NEI genes than those non-DEGs did in the background network (P < 0.001, Kolmogorov-Smirnov test). Moreover, the average distance of DEG-NEI was significantly shorter than that of NEI-NEI (P < 0.001, Kolmogorov-Smirnov test). On the contrary, ASGs and targets of DELRs presented significant longer distance and lower connectivity to NEI genes (P < 0.001, Kolmogorov-Smirnov test). The above different topological features suggested that DEGs of obese adipose tissue pressed much closer to physiologic perturbations in NEI system than DELRs and ASGs did.

### Core Network of Obesity Reveals uc001kfc.1 as Regulator to PI3K Pathway in Adipose Tissue

**Figure 3:**
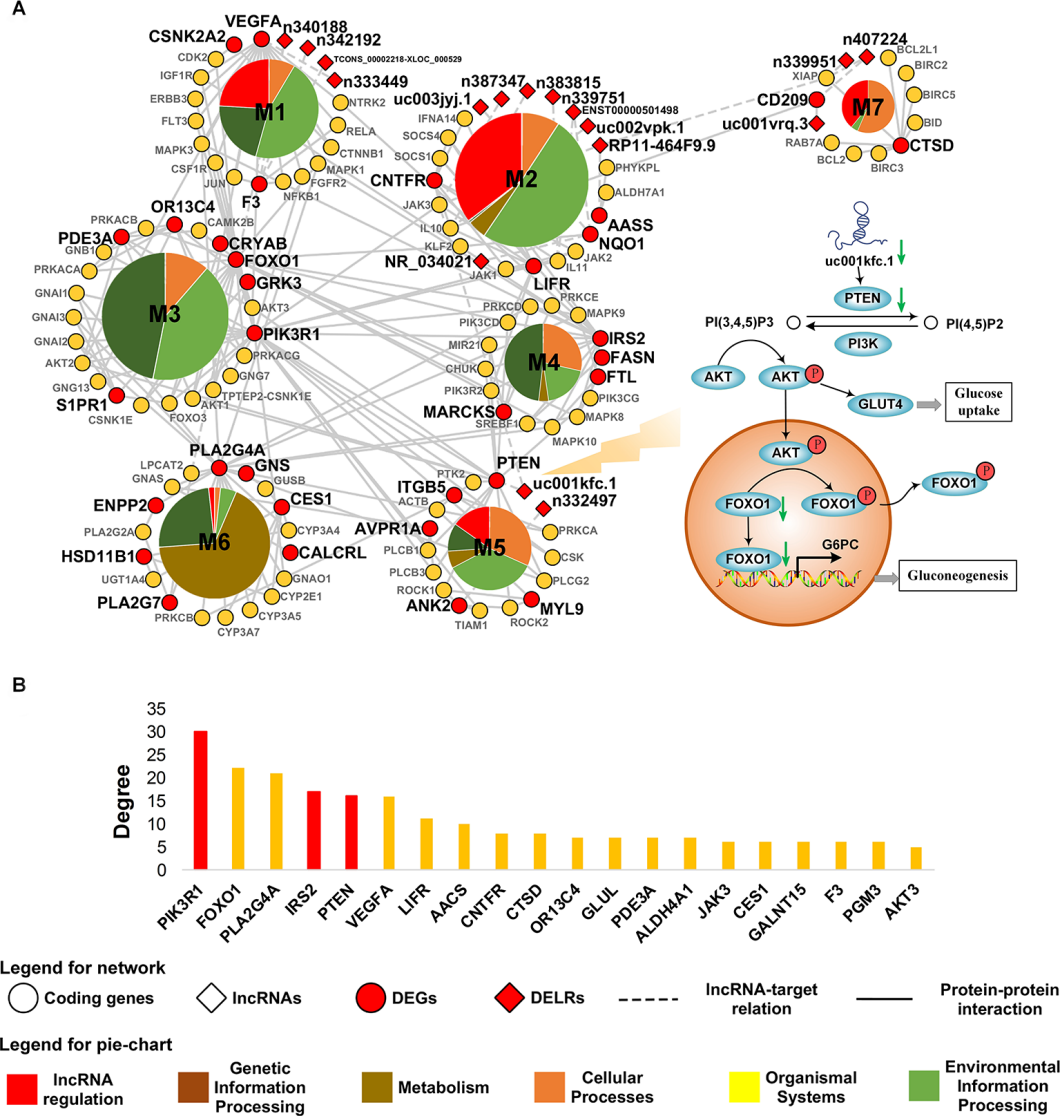
Core network of obese adipose tissue. **(A)** The most connected component in core network of obesity. Biological functions of connections within each module are classified according to the hierarchical sort of pathways in Kyoto Encyclopedia of Genes and Genomes pathway database. **(B)** The TOP20 genes with highest degree in the core network. The bars highlighted by red color denote genes related to PI3K pathway.

Aiming to characterize the core mechanism presented by transcriptome in obesity, a minimized network was constructed based on the altered transcripts in adipose tissue. The largest component of this network was subsequently extracted as the core network of obese adipose tissue because it undertook the main function of a network. To improve its interpretability, the core net was decomposed and exhibited in **Figure 3A**. As it was shown in **Figure 3A**, five out of seven modules in the core network were regulated by DELRs, suggesting the significant role of lncRNAs in obesity. The remaining two modules, M3 and M4, possessed almost half (48.8%) of *inter*-connections across different modules (see **Supplementary Table 3**) and hence could be regarded as hub of the core network. The functions of DEGs in M3 and M4 could be mainly characterized as FoxO and insulin signaling pathways, both of which were well known glucose metabolism regulatory pathways.

A total of 37 DEGs were contained in the core network. To measure their contribution to the connection in the core net of obesity, degrees of genes were measured by calculating how many other nodes that a gene might connect with. **Figure 3B** showed the top 20 of DEGs with highest degree in the core network. As it was highlighted in **Figure 3B**, the top six most connected DEGs were dominated by three genes related to phosphatidylinositol 3-kinase (PI3K) pathway, which was revolved around by FoxO and insulin signaling pathways that were observed in hub modules. Additionally, there were 17 DELRs in the core network and 12 of them were predicted to be able to regulate DEGs (see **Supplementary Table 4**). Among these DELRs, a lncRNA named uc001kfc.1 was predicted to be able to *cis*-regulate a gene directly participating in the PI3K pathway, PTEN. Notably, of all the seven target DEGs, PTEN also possessed the most connections in the core network (see **Supplementary Table 4**). It was indicated that uc001kfc.1 might be a vital regulator to glucose metabolism in obese adipose tissue by potentially targeting PTEN in PI3K pathway.

### Quantitative Real Time Polymerase Chain Reaction Validated Down-Regulation of uc001kfc.1 in Independent Testing Samples

**Figure 4:**
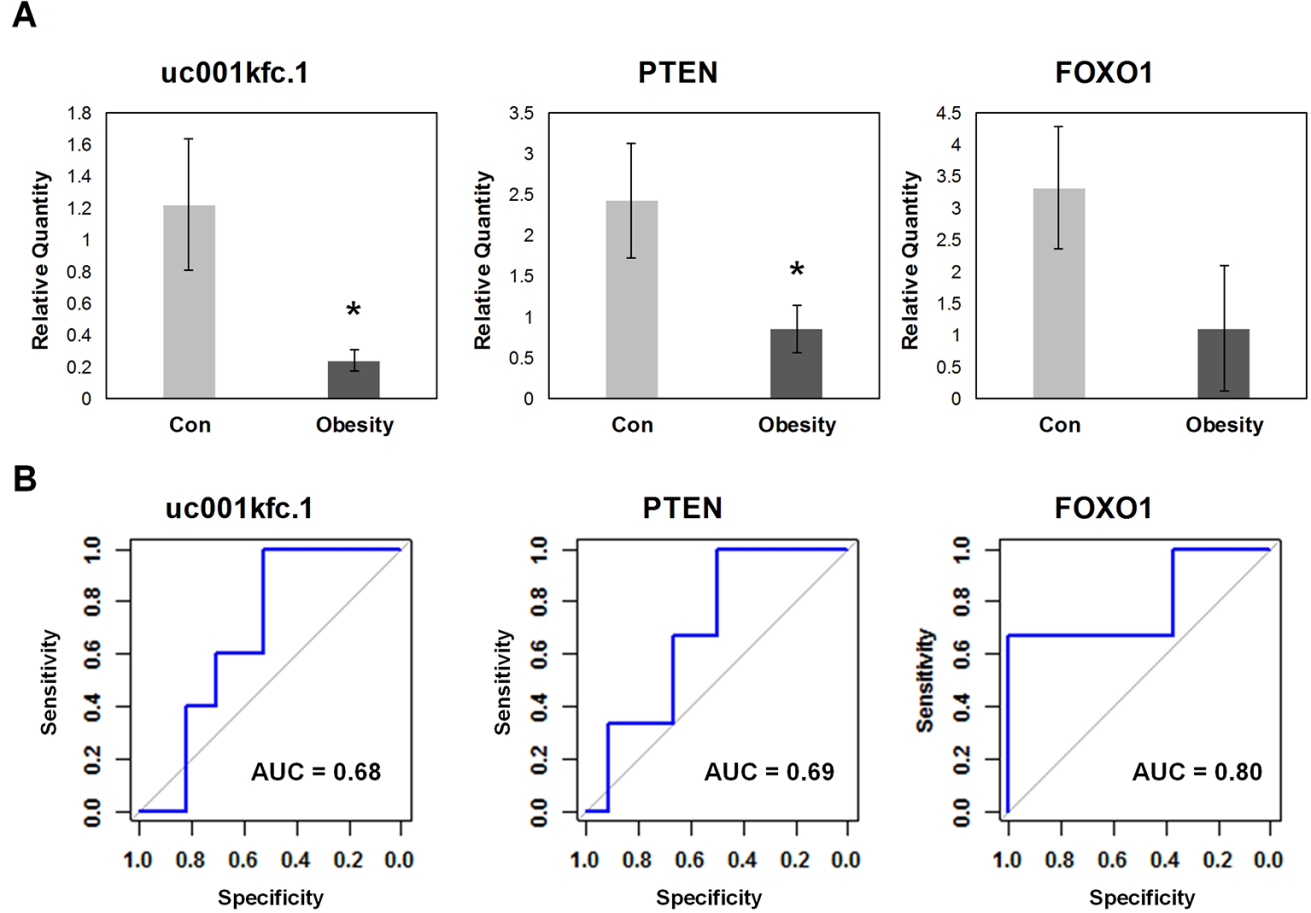
Quantitative real-time polymerase chain reaction validation for candidate genes. **(A)** Relative quantity of candidate genes. *A significant difference between obese and control group exists if p-value 0.05 (Student’s t test). **(B)** Receiver operating characteristic curve of candidate genes.

In order to validate the candidate genes important to glucose metabolism in obesity, expression levels of altered transcripts were further examined by quantitative real time PCR assay in the independent testing sample set including 5 obese subjects and 17 controls. The relative quantity and receiver operating characteristic (ROC) curves of uc001kfc.1, PTEN, and FOXO1 were exhibited in **Figure 4**. Obesity group showed significantly lower expression level of uc001kfc.1 and PTEN than control group (P = 0.016 and 0.028, Student’s t test), which was consistent with results from microarray (FC = −4.74 and −3.66, P = 0.021 and 0.030, one-way ANOVA). Their downstream protein, FOXO1 (FC = −1.51, P = 0.015, one-way ANOVA), was validated to be down regulated in obesity group as well although the difference did not reach to significant level (P = 0.079, Student’s t test). But it was worth noting that FOXO1 showed the best performance (AUC = 0.8) for discrimination of obese and control subjects in testing sample set.

## Discussion

LncRNAs are newly proposed key factors controlling brown adipogenesis and development, but their regulatory effect to white adipocyte is still merely understood. Deciphering the underlying mechanism could be a novel way to discovering potential targets of obesity. Hence, we present the first comprehensive analysis for whole transcriptome of visceral fat tissue from obese patients.

From network’s point of view, we systematically compared three main kinds of transcripts, ASGs, targets of DELRs, and DEGs, in a functional framework, which filled the gap in adipose transcriptome analysis. Unexpectedly, only DEGs showed closer proximity to NEI genes, which was the physiological basis of obesity. It was suggested that coding genes might approach NEI system in a more precise manner than alternative splicing genes and lncRNAs did. This result may be limited to the inherent ability of the prediction process of ASGs and lncRNA’s targets and could be complemented by abundant accumulation of these molecules from wet experiments in the near future. Accordingly, to precisely deciphering mechanism hidden behind transcriptome, we only use DEGs as seed genes to start obesity’s core network construction.

Abnormal glucose homeostasis is one of the common complicating disorders for obesity (Chen et al., 2017). Other than specific metabolic pathways, regulation of glucose metabolism were highlighted in this study. Two typical regulatory pathways of glucose metabolism, FoxO and insulin signaling pathways, were found to be simultaneously enriched by different kind of transcripts. What’s more, they even dominated main function of hub modules in the core network, indicating significant contribution of them to the disturbed glycometabolism. PI3K pathway is another worth noting pathway due to its high proportion (3/6) in TOP6 genes with highest degree in the core net. It was not only one of the most typical path for regulating glucose metabolism (Huang et al., 2018), but also revolved around by FoxO and insulin signaling pathways as basic functional part of them.

When searching for potential lncRNAs accounting for the above perturbated processes in the core network, we found that lncRNA participated most (5/7) functional modules. It was suggested that lncRNA might play an extensive role in pathology of obese adipose tissue. Enthusiastically, among the 17 DELRs in the core network of obesity, only uc001kfc.1 was predicted as a potential regulatory lncRNA to PTEN, the diphosphatase of phosphatidylinositol 3,4,5-trisphosphate (PIP3). Therefore, we proposed a hypothesis as **Figure 3A** showed: in obesity group, decreased uc001kfc.1 might be linked to down-regulation of PTEN, then the subsequently increased PIP3 could phosphorylate and activate serine/threonine protein kinase (AKT). The activated AKT not only can promote glucose uptake by helping glucose transporter type 4 (GLUT4) translocate to plasma membrane (Beg et al., 2017), but also depress gluconeogenesis *via* phosphorylating FOXO1 which would be immediately exported from nucleus (Pan et al., 2017).

To date, uc001kfc.1 (n381526, NONHSAT015462.2) has been only reported in two literatures about cholesteatoma (Gao et al., 2018) and purpura nephritis (Pang et al., 2019). According to NONCODE, this lncRNA is highly expressed in human adipose tissue and no homologues in other non-human animal models has been reported to date (Fang et al., 2018). We identified decreased uc001kfc.1 in obesity group by whole transcriptome analysis and confirmed it in an independent testing sample set *via* qPCR assay. The *cis*-target of this lncRNA, PTEN, was demonstrated to be down-regulated in both microarray and testing samples, which was consistent with previous observation in liver of high fat diet induced obese mice (Ji et al., 2015). Interestingly, mice with adipocyte-specific elimination of PTEN have been observed to gain more weight than wild type but retain improved insulin sensitivity (Morley et al., 2015). Besides, PTEN haploinsufficiency has been proved to be obesogenic but decrease the risk of type 2 diabetes (T2D) owing to enhanced insulin sensitivity (Pal et al., 2012). The contrary effect of PTEN to obesity and T2D may be an explanation to our observation of decreased FBG in obese subjects. Parallelly, as downstream protein of PI3K pathway and transcription factor of G6PC (Valenti et al., 2008), FOXO1 was detected to be decreased in obesity group and confirmed *via* qPCR with AUC = 0.8. This was in accordance with previous observation of down-regulated FOXO1 in adipose tissue of T2D patients (Rajan et al., 2016). The above results together suggested enhanced insulin sensitivity in obese adipose tissue, which was conflict with previous findings about co-occurrence of obesity and impaired glucose regulation (IGR) in populations (Das et al., 2014; Akter et al., 2017). It may be related to the pathological stage and the metabolic status of selected subjects.

Due to the invasive sampling means and cooperation of participants, we only collected 11 obese and 22 control subjects in this study. Although there were more control subjects than obese ones, the obesity rate was still higher than the prevalence of obesity in China (15.7%) and the reported metabolic syndrome rate (24%) in Chinese with gallstone (Zhu et al., 2016; Lu et al., 2017). To obtain reliable results from microarray based on limited samples, we used Tukey’s biweight algorithm which was robust to outliers for differential expression analysis. Meanwhile, a training and testing strategy was designed for independent validation of identified transcripts, which further guaranteed the reliability of our study. As we can look forward to, the identified lncRNA and its mechanism would shed new light on target development of obesity once it is examined in larger cohorts and gain-/loss-of function experiments.

In summary, the present study concentrated on searching for critical transcripts and potential mechanisms in obese adipose tissue from a systematical perspective. By network analysis of whole transcriptome, our work identified decreased uc001kfc.1 as a potential lncRNA *cis*-regulating PTEN to enhance insulin sensitivity of white adipocytes in obese patients. Although abnormal glycometabolism have been revealed in animal models and cohort studies of obesity, our observations contributed new importance to the changes of glucose hemostasis in obese adipose tissue. The mechanism proposed in this study may ultimately help to develop new therapeutic interventions for the treatment of obesity.

## Conclusion

It was recently highlighted that lncRNA played essential role in fat biology. Here, to explore the underlying mechanism of lncRNAs in obesity, we conducted a whole transcriptome analysis in white adipose tissues from obese patients for the first time. All the three kinds of transcripts, DEGs, targets of DELRs, and ASGs were compared in a functional framework based on a global gene regulatory network. They all indicated abnormal glucose metabolism in obesity group while only DEGs showed closer proximity to NEI system. Hence, a lncRNA-regulated core network was constructed by using DEGs as seed nodes. From the core network, we identified a decreased lncRNA, uc001kfc.1, as potential *cis*-regulator for PTEN to enhance insulin sensitivity of white adipocytes in obese patients.

Although whether uc001kfc.1 correlates with risk of T2D deserves to be examined in an expanded cohort with long-term follow-up visit, the present study provides novel insights into understanding the glucose homeostasis in obese white adipocyte. With further improvement, the network-based analyzing protocol proposed in this study would pave an alternative but interesting way to deciphering function of more lncRNAs from other whole transcriptome data.

## Data Availability Statement

The microarray files are deposited at the Gene Expression Omnibus (GEO) database and are available under the accession number: GSE133786.

## Ethics Statement

The present study involving human participants was reviewed and approved by Institutional Review Board of Hebei General Hospital. All participants provided their written informed consent to participate in this study.

## Author Contributions

LY designed the studies, carried out the research, interpreted the results, and wrote the manuscript. XW assisted in data analysis and revised the manuscript. HG and WZ performed the sample preparation and data acquisition. WW assisted in data analysis. HM designed the study, analyzed the data, reviewed and revised the manuscript, and is responsible for the integrity of this work. All authors approved the final version of the manuscript.

## Funding

This work was supported by grants from National Natural Science Foundation of China (81200638), National Natural Science Foundation of Hebei, China (C2019307081), Medical Scientific Research Project of Hebei, China (20190222, 20180019), and Government Funded Program for Clinical Medicine Talent Training and Basic Research Project of Hebei, China (2017).

## Conflict of Interest

The authors declare that the research was conducted in the absence of any commercial or financial relationships that could be construed as a potential conflict of interest.
