# Supplementary material for: Whole Transcriptome Analysis of Obese Adipose Tissue Suggests u001kfc.1 as a Potential Regulator to Glucose Homeostasis
Source: Front Genet. 2019 Nov 21;10:1133. doi: 10.3389/fgene.2019.01133 (PMC6881462; doi:10.3389/fgene.2019.01133)
Supplement: Supplementary file 1 [file Table_1.docx]

Supplementary Material

# Supplementary Method

**RNA extraction and purification.** Per 100mg Adipose sample was homogenized in 1mL of RNAiso Plus by using a homogenizer. Total RNA was extracted using TRIZOL Reagent (Cat#15596-018，Life technologies, Carlsbad, CA, US)following the manufacturer’s instructions and checked for a RIN number to inspect RNA integrity by an Agilent Bioanalyzer 2100 (Agilent technologies, Santa Clara, CA, US).Qualified total RNA was further purified by RNeasy microi kit (Cat#74004, QIAGEN, GmBH, Germany) and RNase-Free DNase Set (Cat#79254, QIAGEN, GmBH, Germany).

**RNA amplification and labeling.** After passing the quality control, total RNA was amplified, labeled and purified by Affymetrix WT PLUS Reagent Kit (Cat#902280, Affymetrix, Santa Clara, CA, US) according to the manufacturer’s instructions to obtain biotin labeled cDNA.

**Array hybridization.** Array hybridization and washing was performed using GeneChip® Hybridization, Wash and Stain Kit (Cat#900720, Affymetrix, Santa Clara, CA, US)in a Hybridization Oven 645 (Cat#00-0331-220V, Affymetrix, Santa Clara, CA, US)and a Fluidics Station 450 (Cat#00-0079, Affymetrix, Santa Clara, CA, US) followed the manufacturer’s instructions.

**Data acquisition.** Arrays were scanned by Affymetrix GeneChip® Scanner 3000 (Cat#00-00213, Affymetrix, Santa Clara, CA, US). Command Console Software (Affymetrix, Santa Clara, CA, US) was used to control the scanner and summarize probe cell intensity data (CEL file generation) with default settings. Then raw data were normalized by Expression Console.

**Hypergeometric cumulative distribution function.** In this article, p-value is defined by a hypergeometric cumulative distribution function to represent the probability of gathering at least *k* desired samples in a group out of a population by chance. It can be calculated by the formula below.

$$\boldsymbol{P-value}\boldsymbol{=1}\boldsymbol{-} \sum_{\boldsymbol{i}\mathbf{=0}}^{\boldsymbol{K-}\boldsymbol{1}} \frac{\boldsymbol{C}_{\boldsymbol{K}}^{\boldsymbol{i}}\boldsymbol{\cdot}\boldsymbol{C}_{\boldsymbol{N-K}}^{\boldsymbol{k-i}}}{\boldsymbol{C}_{\boldsymbol{N}}^{\boldsymbol{k}}}$$

*N*: total number of genes in all human pathways of KEGG PATHWAY database.

*K*: total number of candidate genes in all human pathways of KEGG PATHWAY database.

*k*: number of genes in one pathway.

*i*: number of candidate genes in one pathway.

**Supplementary Table 1. Sequences of primers of quantitative PCR**

| **Genes** | **Primer sequence** |
| --- | --- |
| FOXO1-Forward | GCAGCCGCCACATTCAACAG |
| FOXO1-Reverse | AGAACTTAACTTCGCGGGGC |
| uc001kfc.1-Forward | TCCTCAAAATTAAAGTGGAAAAGGA |
| uc001kfc.1-Reverse | AATTAAAGCTAATAAGGGCCAGTTT |
| PTEN-Forward | TTCCAAGAAAGCATGCCACAG |
| PTEN-Reverse | TGCAGATAGTGTGGAGCACAG |

**Supplementary Table 2. Demographic information of the microarray samples.**

|  | Control Group (*n* = 17) **^§^** | Obesity Group (*n* = 5) **^§^** |
| --- | --- | --- |
| Age (Years) | 40.2 ± 12.3 | 55.5 ± 11.8 |
| Gender (Male, %) | 3 (60%) | 2 (33.3%) |
| Waistline (cm) | 85.6 ± 9.2 | 111.4 ± 13.6***** |
| Hipline (cm) | 97.8 ± 5.8 | 118.3 ± 14.6***** |
| Height (cm) | 165.4 ± 7.0 | 161.8 ± 8.8 |
| Weight (kg) | 63.2 ± 7.4 | 88.8 ± 6.5***** |
| BMI (kg/m^2^) | 23.2 ± 2.9 | 34.1 ± 3.6***** |
| TC (mmol/L) | 4.22 ± 0.21 | 4.71 ± 0.56 |
| TG (mmol/L) | 0.72 ± 0.34 | 1.95 ± 1.25 |
| HDL-C (mmol/L) | 1.29 ± 0.25 | 1.47 ± 0.89 |
| LDL-C (mmol/L) | 2.67 ± 0.41 | 2.77 ± 0.90 |
| FBG (mmol/L) | 5.29 (4.89-5.56) | 5.17 (5.07~10.65) |

**^§^**Information is separately presented as *n* (%) for dichotomous data, median (min ~ max) for non-normal data and mean±sd for normal data.

*A significant difference between obesity and control group exists if p-value < 0.01 (two tailed Student’s t test).

^#^ A significant difference between obesity and control group exists if p-value < 0.01 (two tailed Kolmogorov-Smirnov test).

**Supplementary Table 3. Number of inter- connections of each module**

| **Module** | **Number of inter- connections with other modules (*n*, %)** |
| --- | --- |
| M3 | 38 (29.5%) |
| M4 | 25 (19.4%) |
| M5 | 20 (15.5%) |
| M1 | 18 (14.0%) |
| M6 | 14 (10.9%) |
| M2 | 12 (9.3%) |
| M7 | 2 (1.5%) |

**Supplementary Table 4. Degrees of differentially expressed targets of DELRs in the core network of obesity**

| **DELR** | **Differentially expressed targets** | **Degree** |
| --- | --- | --- |
| uc001kfc.1 | PTEN | 16 |
| n333449 | VEGFA | 16 |
| n340188 | F3 | 6 |
| n342192 | F3 | 6 |
| TCONS_00002218-XLOC_000529 | F3 | 6 |
| n332497 | ANK2 | 4 |
| uc003jyj.1 | NQO1 | 4 |
| ENST00000501498 | NQO1 | 4 |
| NR_034021 | NQO1 | 4 |
| RP11-464F9.9 | AASS | 3 |
| uc001vrq.3 | CD209 | 3 |
| n407224 | CD209 | 3 |
